# Effects of steroid growth promoter on morphological and biochemical adaptations in liver of broiler

**DOI:** 10.14202/vetworld.2020.2330-2337

**Published:** 2020-11-05

**Authors:** Nasrin Sultana, Marzia Afrose, Kazi Rafiq

**Affiliations:** 1 Department of Anatomy and Histology, Faculty of Veterinary Science, Bangladesh Agricultural University, Mymensingh, Bangladesh; 2 Department of Pharmacology, Faculty of Veterinary Science, Bangladesh Agricultural University, Mymensingh, Bangladesh

**Keywords:** broiler, dexamethasone, growth promoter, liver

## Abstract

**Aim:**

The study was conducted to observe the effects of dexamethasone (DEX) on the gross study and histomorphometry of liver and on the alterations of biochemical parameters of broilers.

**Materials and Methods:**

Ninety day-old chicks were collected and assigned to one of three groups: The control, Group A, and Group B. The control, Group A, and Group B were fed for 28 days with a homemade ration, a commercial broiler type ration, and a homemade ration with DEX (7 mg/kg feed), respectively. Liver samples were collected from the individual birds after sacrificing on days 7, 14, 21, and 28 of the experiment. Morphometric characteristics (length, weight, color, and texture) of the liver were examined. Histomorphological alterations of the liver were assessed with routine hematoxylin and eosin staining. To measure the biochemical parameters, blood samples were collected on days 7, 14, 21, and 28 of the experiment. Liver function test was performed spectrophotometrically by analyzing serum biochemical markers, that is, alanine aminotransferase (ALT), aspartate aminotransferase, and alkaline phosphatase (ALP). Thin-layer chromatography (TLC) was performed for the detection of hepatic steroids.

**Results:**

Hemorrhagic and congested livers were found in broilers of Group B. There were no significant changes found in weight and length of the livers; only numerical decrease in weight and length was observed in birds of Group B. Liver width was increased in Group B on day 21. Histological observation of livers showed accumulation of lipid droplets, congestion of the sinusoids, and central veins in broilers of Group B. Biochemical analyses showed increased levels of ALT in Group B as compared to Group A on day 14 of the experiment. TLC evaluation revealed a positive result in Group B on 28 days of the experiment.

**Conclusion:**

The present study results show that DEX may alter the liver morphology and the concentration of ALT in the circulation of broilers.

## Introduction

Nowadays, the poultry sector is becoming a leading industry among agriculture in Bangladesh. For the past two decades, this sector has been growing by an annual rate of around 35%[1]. This industry has immense potential for boosting the economic growth of the country as well as ensuring food security. A joint IFPRI/FAO/ILRI study suggested that global production and consumption of meat will continue to rise from 233 million metric tons (MT) in the year 2000 to 300 million MT in 2020, with particularly poultry meat production growing from 9 MT in 1960 to 68 MT in 2000 due to increasing national demand [2]. This has triggered the discovery and widespread use of a number of “growth promoters (GPs).” The GPs are added to the ration with the purpose of boosting animal performance by increasing the growth rate, improving the feed conversion efficiency, increasing survivability, and lowering mortality in poultry birds [3]. Further, GPs are gaining popularity as feed additives due to their favorable effects on gut health and immunity [4,5].

Glucocorticoids (GCs) are steroid hormones containing both natural and synthetic derivatives [6]. More than 50 steroids have been isolated from the adrenal cortex of different species [7]. Only a few steroids are primarily responsible for the effects of the adrenal cortex on carbohydrate, protein, and fat metabolism. As such, the effects mediated by GCs are for instance increased gluconeogenesis, decreased peripheral glucose utilization, increased insulin antagonism, increased protein catabolism, reduced fat storage, and stimulated anti-inflammatory effects [8], including reduced circulatory lymphocytes, eosinophils, and fixed lymphocytic tissues and decreased local inflammatory processes [9]. Dexamethasone (DEX) is a synthetic derivative of GCs, whose major therapeutic usage derives from its anti-inflammatory and immunosuppressant properties [8]. A large proportion of DEX used in veterinary practices is designated to combat inflammation or allergy. Therefore, misuse of that corticosteroid is not uncommon in this category. In meat cattle industry, DEX is commonly abused for increasing the growth performance of cattle [6,10,11]. Illegal use of steroid GP at a high dose, that is, 7mg/kg [12] and their residues in food products may negatively impact children’s mental and physical growth and women’s fertility, cause cancer, and damage vital organs such as brain, liver, kidney, and heart [13]. Due to these health concerns, some of the steroid GPs were banned from using in food-producing animals by the European Union (EU) [14,15]. To protect the consumer health from any potential risks, the EU approved the use of DEX in livestock for therapeutic purposes only and set appropriate maximal residue limits in tissues and milk intended for human consumption (Council of the European Communities 1990, Council Regulation 90/2377/EEC) [14].

The liver, the largest gland of the body, is used to filter harmful toxins from the blood and for the synthesis of vitamins and minerals. It is also important for balancing levels of proteins, cholesterol, and sugars. Further, the liver is used for the production of bile aiding in the digestion of food. In general, GCs may cause immune suppression leading to non-specific bacterial, viral, and parasitic growth in the body and toxins of these microbes may also create liver damage. The GCs are enzyme and heat resistant and are commonly deposited in the liver [16] resulting in hepatotoxicity. Due to hepatotoxicity, some liver-specific enzymes (alanine aminotransferase [ALT], aspartate aminotransferase [AST], and alkaline phosphatase [ALP]) may be released in blood. In general, increased circulating enzyme concentrations are indicators of acute organ damage rather than decreased organ function. Increase of plasma AST and ALT concentrations is the most specific indicators of liver cell damage [17].

The present study aimed to observe the histomorphology of liver and the alterations of biochemical parameters in blood of broilers in response to steroid GP DEX.

## Materials and Methods

### Ethical approval

All animal handling procedures were in compliance with the care and use of rules as established by Animal Welfare and Experimentation Ethics Committee, Bangladesh Agricultural Committee, Mymensingh (AWEEC/BAU/2019[23]).

### Birds and management

The present experiment was conducted in January 2019 in the Department of Anatomy and Histology, Bangladesh Agricultural University, Mymensingh-2202. Ninety day-old chicks (DOCs) of a “Cobb 500” strain were considered for the experiment. They had been reared in an environmentally controlled room for 28 days at the experimental shed. The broilers were fed with standard broiler starter and broiler finisher ration throughout the experimental period.

### Experimental design

A total of 90 DOC “Cobb 500” broilers were randomly divided into three equal groups (each n=30): Control group, Group A, and Group B. The birds of the control group, Group A, and Group B were reared on homemade broiler ration, a commercial type of broiler ration, and homemade broiler ration with a high dose of DEX, respectively. Blood samples from the wing vein and liver samples were collected on days 7, 14, 21, and 28 of the experimental period. The liver samples were aseptically removed from each broiler for gross observation, histological study, and thin-layer chromatography (TLC).

### Experimental diet

The birds of the control group were reared on homemade broiler ration. The broilers of Group A were reared with a commercial type of broiler ration. The broilers of Group B were reared with homemade broiler ration including a high dose of DEX (7.0 mg/kg body weight) supplementation in the feed. The commercial ration was brought from the Nourish Poultry and Hatchery Ltd., Bangladesh. The homemade ration was formulated according to the ingredients of commercial ration (Table-1). Each ingredient of the ration was prepared separately and then mixed together in the homemade diet.

**Table-1:** Feed ingredients and chemical composition of ration.

Ingredients	Broiler pre-starter	Broiler starter	Broiler finisher
Maize	43.00 kg	40.32 kg	43.64 kg
Wheat	10.00 kg	10.00 kg	10.00 kg
Rice polish	4.00 kg	8.00 kg	10.00 kg
Soybean	26.00 kg	29.00 kg	22.50 kg
Meat and Bone meal	9.00 kg	7.00 kg	8.00 kg
Oyster shell	1.00 kg	1.00 kg	1.00 kg
Salt	300 g	300 kg	250 g
Methionine	200 g	200 g	180 g
Lysine	30 g	30 g	30 g
Vitamin premix (broiler)	250 g	250 g	250 g
Feed enzyme	-	-	50 g
Soybean oil	6.5 kg	3.5 kg	4.0 kg
DCP	2.50 kg	2.50 kg	-
Choline chloride	100 g	100 g	100 g
Total	100.00 kg	100.00 kg	100.00 kg

### Gross study

In the gross study, parameters such as color, length, and weight were taken into consideration. The color of the liver was compared within the control and experimental groups by visual observation. Length and width of the liver of the different groups were measured by a graded scale.

### Liver histology

The liver samples were rapidly removed and preserved in 10% neutral buffered formalin. After a 24 h fixation period, the tissues were dehydrated and then embedded in paraffin. The paraffin blocks were cut into 6 μm thick sections by a sliding microtome (MIC 509, Euromex, Japan). The samples were floated onto glass slides, dried overnight, and then stained with hematoxylin and eosin to determine the morphological changes.

### Biochemical studies

Five milliliters of blood were collected without anticoagulant in sterile glass test tubes. The blood containing tubes were placed in a slanting position at room temperature for clotting. The tubes were then placed in the refrigerator at 4°C overnight. Blood was centrifuged at 1000 rpm for 15 min for serum collection. The serum was then stored in a screw-capped vial and preserved at −20°C until further use. The measurement of biochemical parameters was performed by spectrophotometer using a Humalyzer 2000 analyzer (Wiesbaden, Germany).

### TLC

The liver samples on day 28 of the different experimental groups were considered for qualitative testing. The samples were first blended with mortar and pestle. Then, PBS (pH6.5) was added and samples were homogenized by a vortex machine (Vortex-XHC, Wincom, China). Following this, 30% trichloroacetic acid was added and the mixture was centrifuged (Hettich D-78532, Germany) at 6000 rpm for 20 min. The supernatant of the fluid was then transferred into a falcon tube, with an equal amount of diethyl ether added and the bottom layer was collected into a screw-capped vial. With a pencil, a thin mark was made at the bottom of the TLC plate to apply the sample spots. Then, the sample solutions were applied on the spots marked on the line in equal distances. The mobile phase was poured into the TLC chamber to a leveled notch a few centimeters above the chamber bottom. A moistened filter paper in the mobile phase was placed on the inner wall of the chamber to maintain equal humidity. The plate prepared with the sample spots was then placed in the TLC chamber so that the side of the plate with the sample line was facing the mobile phase and the chamber was closed with a lid. The plate was immersed so that the sample spots were well above the level of the mobile phase for development. The plates were removed and allowed to dry at RT. The sample spots were detected in a suitable UV light chamber.

### Statistical analysis

The data obtained from the measurement of weight, length and width of liver, and biochemical parameters (ALT, AST, and ALP) were analyzed using the repeated measurement procedure by the Statistical Package for the Social Sciences software version 19 (IBM Corp., NY, USA). Differences among the mean were identified by Duncan’s multiple range tests. The level of significance was set at p<0.05.

## Results

### Gross observation

#### Gross appearance of liver

**Figure-1:**
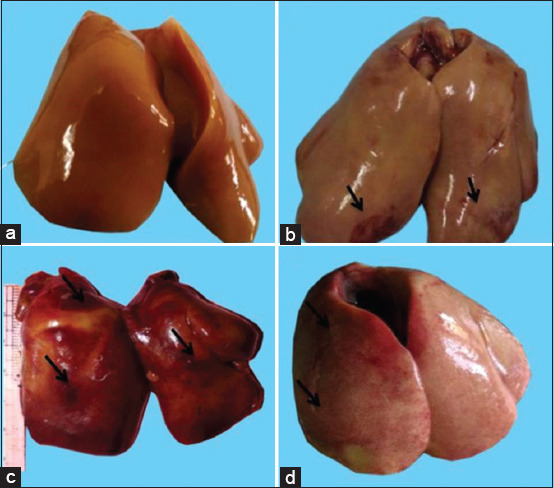
Representative images of gross observation of liver of different groups of broiler. Normal appearance of liver in the control group (a), congested (black arrow) liver in Group A (b), and both congested (c) and hemorrhagic (d) liver were seen in dexamethasone-treated group, that is, Group B.

There were no gross changes observed in the liver of broilers of the control group. The color of the liver was found to be reddish in the control group whereas it was pale in color in broilers of Group A. Congestion was found in the liver of broilers of Group A on 21 and 28 days of the experiment (Figure-1). Both the hemorrhagic and congested livers were found in broilers of Group B (Figure-1a) on days 14, 21, and 28 of the experiment.

#### Liver weight

**Figure-2:**
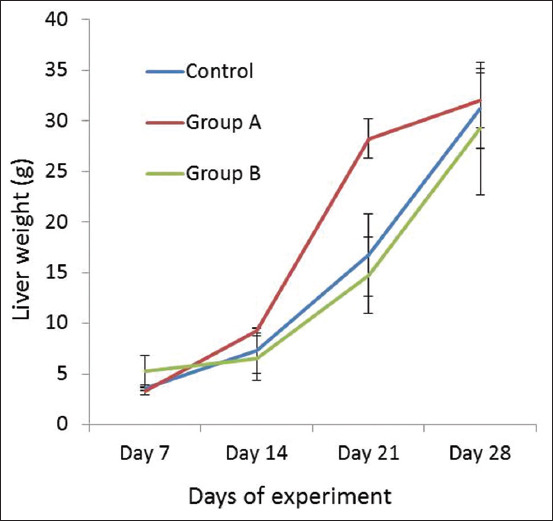
Weight of liver of broiler in different experimental groups on different days of experimental period.

The mean weight of livers in the control group, Group A, and Group B on day 7 was 3.63±0.24 g, 3.33±0.42 g, and 5.25±1.59 g, respectively (Figure-2). On day 14, the highest weight was observed in Group A (9.25±0.25 g) compared with the control group (7.3±2.23 g) and Group B (6.55±2.21 g). On day 21 of the experiment, lowest liver weight was recorded in Group B (14.75±3.75 g) compared to Group A (28.75±1.93 g). The mean weight of livers in the control group, Group A, and Group B on day 28 was 31.25±3.94 g, 32±2.68g, and 29.25±6.54 g, respectively.

#### Liver length

**Figure-3:**
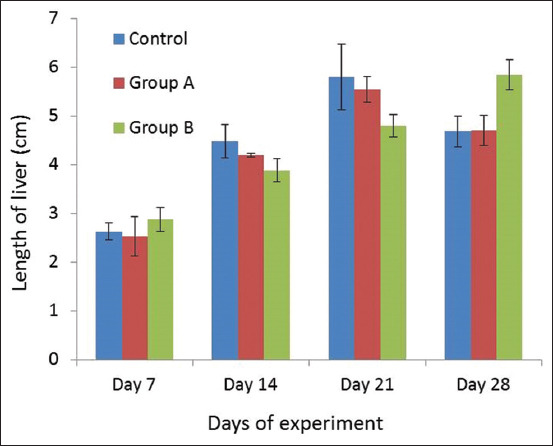
Length of liver in different experimental groups of broiler on different days of experimental period (Mean±SE).

The length of liver is shown in Figure-3. There were no significant differences observed among the different groups of broiler chicken. Only a numerical decrease was observed in liver length of broilers in Group B on days 14 and 21 of the experiment.

#### Liver width

**Figure-4:**
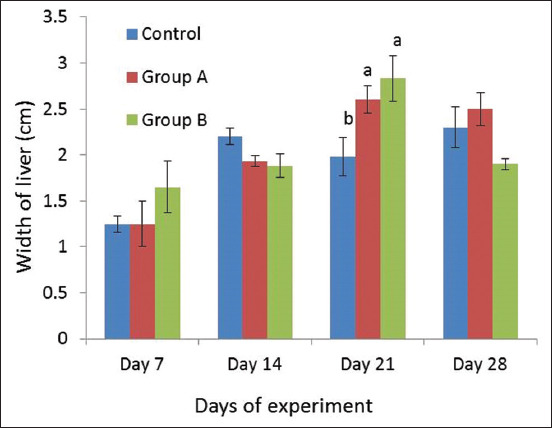
Width of liver (Mean±SE) of broiler of different experimental groups on different days of experiment. Different letters as superscript indicate the significant differences between the different groups of experiment.

The width of liver of three experimental groups is presented in Figure-4. We observed higher mean values in broilers of Group B on day 21 of the experiment as compared to control and Group A.

### Histological observation

**Figure-5:**
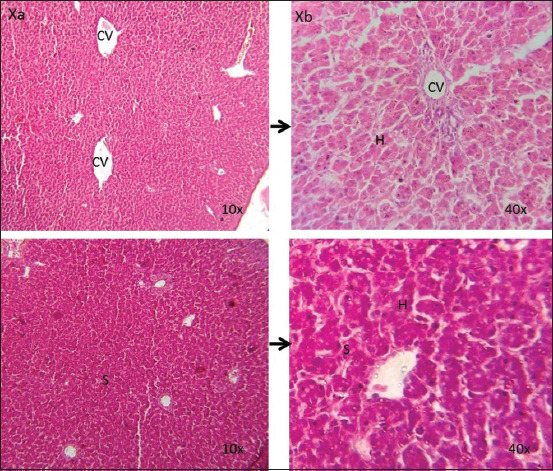
Photomicrographic representation of liver sections in the control group (10× and 40×). Liver shows the normal histological architecture (Xa and Xb) (H and E). CV=Central vein; S=Sinusoids; H=Hepatocytes.

**Figure-6:**
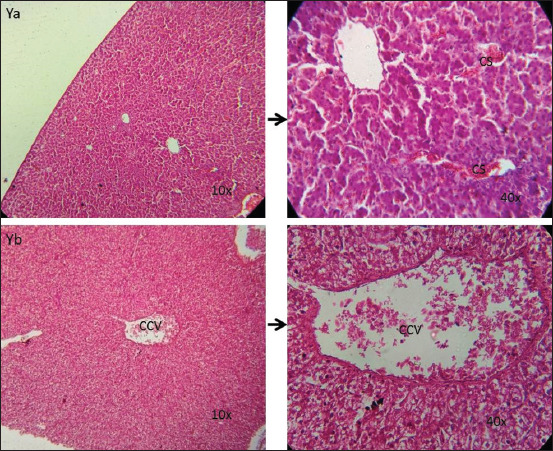
Photomicrographic representation of liver sections in broilers of Group A (10× and 40×). Group A showing the sinusoidal congestion (Ya), dilated and congested central vein (Yb) on 21 and 28 days of liver sections (H and E).CCV=Congested central vein; CS=Congested sinusoids.

**Figure-7:**
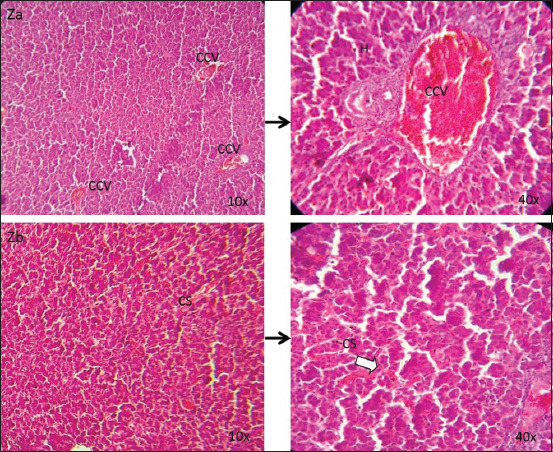
Photomicrographic observation of liver sections in Group B (10×, 40×). Group B showing the dilated and congested central vein (Za) and congested sinusoids (white arrow) (Zb) on 21 and 28 days of liver sections (H and E). CCV=Congested central vein; CS=Congested sinusoids; H=Hepatocytes.

**Figure-8:**
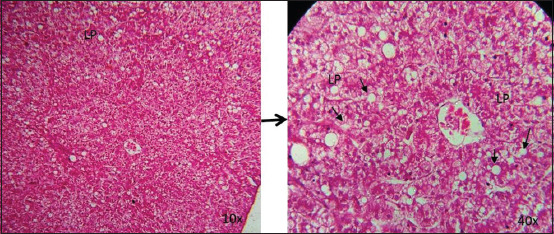
Photomicrographic representation of liver sections in Group B (10×, 40×). Lipid droplets (black arrow) were seen in the liver sections (Zc) on day 28 in Group B (H and E). CV=Central vein; LP=Lipid droplets.

The liver of the control group was found with normal histological architecture at all time points (Figure-5). On days 21 and 28, congestions in the sinusoids and central vein were detected in the liver sections of Group A (Figure-6), whereas at those time points, congestions in the central vein and sinusoids as well as accumulation of fat droplets were observed in Group B (Figures-7 and 8).

### Biochemical analysis

**Figure-9:**
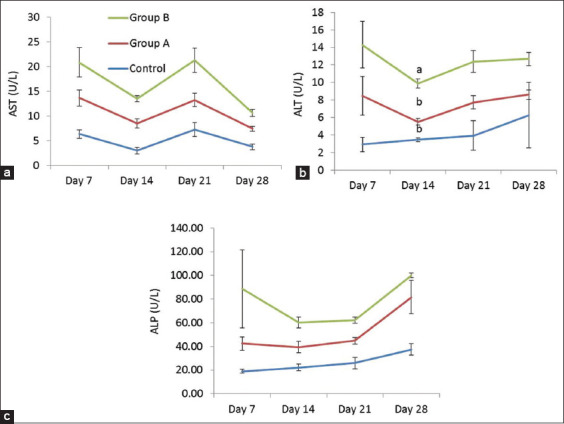
(a-c) Effects of steroid growth promoter on the measurement of liver enzymes in serum of different groups of broiler (Mean±SE). Different letters as superscript indicate the significant differences between the different groups of experiment.

In the present study, liver function test was performed by measuring the serum level of three different biochemical parameters, that is, ALT, AST, and ALP in three experimental groups on days 7, 14, 21, and 28 (Figure-9). The level of ALT was significantly increased in the serum of Group B on day 14 as compared to Group A, whereas the level of AST and ALP remained unchanged in broilers of all groups.

### TLC

**Figure-10:**
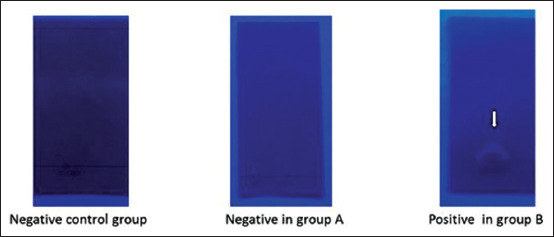
Qualitative identification of the steroid substances was made by thin-layer chromatography.

TLC is a solid-liquid form of chromatography that can be used to verify a substance’s identity. In the liver samples, qualitative identification of the DEX was made by TLC (Figure-10). The positive control was the DEX-treated group, that is, Group B; negative results were showed by the control group and Group A which was maintained by commercial broiler-type ration showing positive results compared to Group B.

## Discussion

In the present study, high doses of DEX were fed to broilers to observe the effects of GC on gross and morphological changes of liver and on alterations of biochemical parameters in serum.

### Body weight

Body weight of broilers in the DEX-treated group was reduced in comparison to the control group and Group A in the same experiment performed by our group [12]. According to the literature [18], DEX may increase plasma T3 levels and stimulate metabolic pathways in muscle, which are responsible for muscular dystrophy. A group of scientists [19] stated that chronic consumption of corticosteroid in the diet may stimulate protein catabolism which can suppress body weight gain and decrease feed intake of broiler chickens.

### Gross observation of liver

The liver of the control group was found reddish in color, whereas slight congestion was found in the liver of Groups A and B. Pinpoint-type hemorrhagic lesions were found in broilers of Group B. Similar findings have also been reported by Dattani and Brook [7] and stating that congestion may be occurred due to micronodular cirrhosis in liver.

In the present study, the lowest liver weight was recorded in Group B. However, liver weight increased with increasing the age of the broilers, which in accordance with results presented in Cai *et al*. [20]. It has been reported that the liver proportion (as % of BW) of chickens with DEX injection increases by 34% of the control and 39% of the pair-fed chickens. Other studies have also observed remarkable increases in the liver mass by corticosterone (CORT) treatment [21-24]. It is probable that increased liver mass can be caused by enhanced fatty acid synthesis and central fat deposits in the liver response to high circulating CORT levels [25,26]. Liver weight results found in our study are opposed to those found in another study conducted by a group of researchers [27].The relative and actual mass of the liver was significantly higher in DEX-treated chickens.

### Histomorphological observations

A normal histological architecture was observed in the liver of the control group. However, congested central veins and sinusoids were found in Group A. In Group B, the congestion was seen in the central vein and sinusoids of the liver on 21 and 28 days of the experimental period. The accumulation of lipid droplets was also observed on day 28 in Group B. Similar results were reported by other groups [28-31] stating that the chronic elevations in GCs may be associated with increased incorporation of liver fat. GCs stimulate hepatic gluconeogenesis and increase the hepatic synthesis and storage of glycogen in the liver [30]. In response to elevated blood glucose, there may be a compensatory increase in insulin secretion. Treatment with DEX can cause time-dependent changes in glucose and insulin levels, and increase in secretion of insulin, leading to more glycogen being deposited in the cytoplasm of hepatocytes overtime. A group of researchers [32] observed a slight cytoplasmic vacuolization identified as lipid droplets, affecting a few hepatocytes. Further, a wide cytoplasmic vacuolization resulting from intracellular edema and lipid accumulation was also observed. DEX inhibits the synthesis of arachidonic acid and prostaglandin which normally act as antiaggregant agents [30]. This, together with the likely occurrence of hypertension and polycythemia in the liver may have caused the sinusoidal dilatation and congestion, which have been noticed in the DEX-treated group of the present study.

### Biochemical parameters

The elevation of ALT, AST, and ALP in serum and tissues is the most common signal of liver diseases [17]. In the present study, the level of ALT was significantly increased in serum of Group B on day 14 of the experiment as compared to Group A. The elevated level of ALT enzyme released into the blood may be due to liver damage considering that ALT is specific for hepatocytic necrosis [33,34].

In the present study, the serum AST and ALP levels in Groups A and B were not significantly different from the control group. It has been shown that DEX treatment may increase ALT and AST levels in serum of broilers at 3 weeks of age; however, there was no significant difference of ALT and AST values in weeks 4, 5, and 6 [35]. A similar report was found in another research [36]. These results on ALT and AST are in the same line to the present research. In a different study, it has been observed that serum levels of ALP, AST, and ALT may be decreased in low-dose DEX and high-dose DEX rats with bile duct ligation due to cholestasis or diffused liver injury [37]. They explained that this alteration may be due to the cholestasis or diffused liver injury.

### Screening method

As TLC has proven to be a successful screening method to assess corticosteroid residues in tissues of different species [9,38], we chose the same method to detect the presence of DEX in our samples. Group B which received the homemade diet with DEX showed a positive result for residues of DEX in the liver by the presence of spots on the chromatographic plate. A similar observation was made by another research group [39] where tissue samples were analyzed by shining ultraviolet light on the plate. As predicted, samples from broilers of the control and Group A showed a negative result. The present study indicates that DEX may deposit in the liver of broilers in response to exogenous GC DEX.

## Conclusion

In gross observation, we observed consistent liver weight in the different groups of broilers during the experiment except for slight decreases in Group B. Only a numerical decrease was observed in the length of liver of Group B, whereas the width of liver was increased in this group. A congested and hemorrhagic liver was seen in Group B. Normal liver morphology was found in the control group, whereas congested central veins and sinusoids were seen in Groups A and B. Lipid droplets were accumulated in liver sections of broilers of Group B on day 28. The level of ALT was significantly increased in serum of Group B. The level of AST and ALP was unchanged in the broilers of the different experimental groups. The screening method revealed the presence of DEX in liver tissues of broilers which were supplemented with DEX. Finally, it was found that DEX alters liver growth and morphology as well as the concentration of ALT in the circulation of broiler chicken. DEX might balance the liver weight by increasing the width and decreasing the length of the liver in broilers.

### Authors’ Contributions

NS: Chief of the research, conceptualized and designed the research, and drafted and revised the manuscript. MA: Conducted the experiment, data collection and analysis, and manuscript drafting. KR: Helped in conducting the experiment and data collection. All authors read and approved the final version of the manuscript.
